# Global transcriptomic characterization of T cells in individuals with chronic HIV-1 infection

**DOI:** 10.1038/s41421-021-00367-x

**Published:** 2022-03-28

**Authors:** Xiang-Ming Wang, Ji-Yuan Zhang, Xudong Xing, Hui-Huang Huang, Peng Xia, Xiao-Peng Dai, Wei Hu, Chao Zhang, Jin-Wen Song, Xing Fan, Feng-Ying Wu, Fu-Hua Liu, Yuehua Ke, Yifan Zhao, Tian-Jun Jiang, Li-Feng Wang, Yan-Mei Jiao, Ruo-Nan Xu, Lei Jin, Ming Shi, Fan Bai, Fu-Sheng Wang

**Affiliations:** 1 Biomedical Pioneering Innovation Center (BIOPIC), School of Life Sciences, Peking University, Beijing, China; 2 Beijing Advanced Innovation Center for Genomics (ICG), Peking University, Beijing, China; 3 Senior Department of Infectious Diseases, the Fifth Medical Center of Chinese PLA General Hospital, National Clinical Research Center for Infectious Diseases, Beijing, China; 4 Center for Disease Control and Prevention of Chinese PLA, Beijing, China

**Keywords:** Autoimmunity, Bioinformatics

## Abstract

To obtain a comprehensive scenario of T cell profiles and synergistic immune responses, we performed single-cell RNA sequencing (scRNA-seq) on the peripheral T cells of 14 individuals with chronic human immunodeficiency virus 1 (HIV-1) infection, including nine treatment-naive (TP) and eight antiretroviral therapy (ART) participants (of whom three were paired with TP cases), and compared the results with four healthy donors (HD). Through analyzing the transcriptional profiles of CD4^+^ and CD8^+^ T cells, coupled with assembled T cell receptor sequences, we observed the significant loss of naive T cells, prolonged inflammation, and increased response to interferon-α in TP individuals, which could be partially restored by ART. Interestingly, we revealed that CD4^+^ and CD8^+^ Effector-GNLY clusters were expanded in TP cases, and persistently increased in ART individuals where they were typically correlated with poor immune restoration. This transcriptional dataset enables a deeper understanding of the pathogenesis of HIV-1 infection and is also a rich resource for developing novel immune targeted therapeutic strategies.

## Introduction

Chronic human immunodeficiency virus type 1 (HIV-1) infection typically results in the progressive loss of CD4^+^ T lymphocytes, and overactivation and functional exhaustion of CD8^+^ T lymphocytes, which can lead to acquired immunodeficiency syndrome (AIDS) if untreated^1–3^. Much attention has been focused on T cell immunology of HIV-1 infection, with research objectives ranging from the classical to newly identified T cell subsets. Specifically, the selective loss of naive CD4^+^ T cells, naive CD8^+^ T cells, and memory CD4^+^ T cells, and the expansion of memory and effector CD8^+^ T cells skew the CD4/CD8 ratio, reflecting host immune disorders caused by chronic HIV-1 infection^2,3^. Aberrant immune responses of T cell subsets have been extensively studied^4,5^. Recently, several novel T cell subsets have been identified^6–10^; for example, systemic loss of mucosal-associated invariant T (MAIT) cells that confer protection to the mucosal barrier is associated with disease progression^11^. However, due to technical limitations, these studies primarily focused on selected T cell subsets. A more comprehensive understanding of the synergistic immunologic responses among the full spectrum of T cell subsets is urgently needed during the natural course and antiretroviral therapy (ART) of the disease.

ART efficiently suppresses viral replication, which indirectly increases CD4^+^ T cell count, improves the CD4/CD8 ratio and repairs immune disorders. However, the degree of host immune system recovery relies on the stage of disease progression and the time ART was started^12–14^. Approximately 20–30% of individuals with HIV-1 infection, namely, immune non-responders (INRs), are unable to achieve normal immune restoration despite successful ART^12,15–19^. INRs often have a high risk of non-AIDS-related morbidity and mortality when compared to immune complete responders (CR)^6,12,20,21^. Up to now, it is not fully understood how the composition and transcriptomic profiles of whole T cell subsets are altered and how these alterations influence T cell immune recovery and clinical outcome in participants during ART.

Recently, single-cell RNA sequencing (scRNA-seq) has been utilized to obtain the whole immune cell landscape of individuals with chronic HIV-1 infection^22^. scRNA-seq yields valuable information on the biology of HIV-1 latency^23,24^ and the mechanisms of inflammation^25^. Critical immune cell subsets have been investigated. For example, TRAIL^+^ innate immune cells were identified as the driving force for CD4^+^ T cell depletion in a humanized mouse model^26^; CD69^+^ tissue-resident memory CD8^+^ T cells in the lymph nodes^6^ and highly functional antiviral dendritic cells (DCs) in the peripheral blood mononuclear cells (PBMCs) were considered superior mediators for controlling HIV-1 infection^27^. In addition, Kazer et al. demonstrated multicellular immune dynamics during hyperacute HIV-1 infection^28^. It is also viable and important to identify the dynamics of T cell subsets during chronic HIV-1 infection and explore their changes following ART with scRNA-seq.

To address the abovementioned issues, we used scRNA-seq, coupled with assembled T cell receptor (TCR) sequences, to comprehensively analyze the immunological characteristics and alterations of T cell subsets during chronic HIV-1 infection. In particular, we compared the composition and transcriptional profiles of T cell subsets before and after ART. Our study provides a high-resolution transcriptomic atlas of T cells, which will facilitate a better understanding of the protective and pathogenic roles of T cells during chronic HIV-1 infection.

## Results

### Transcriptional profiling of T cells in individuals with chronic HIV-1 infection

**Fig. 1:**
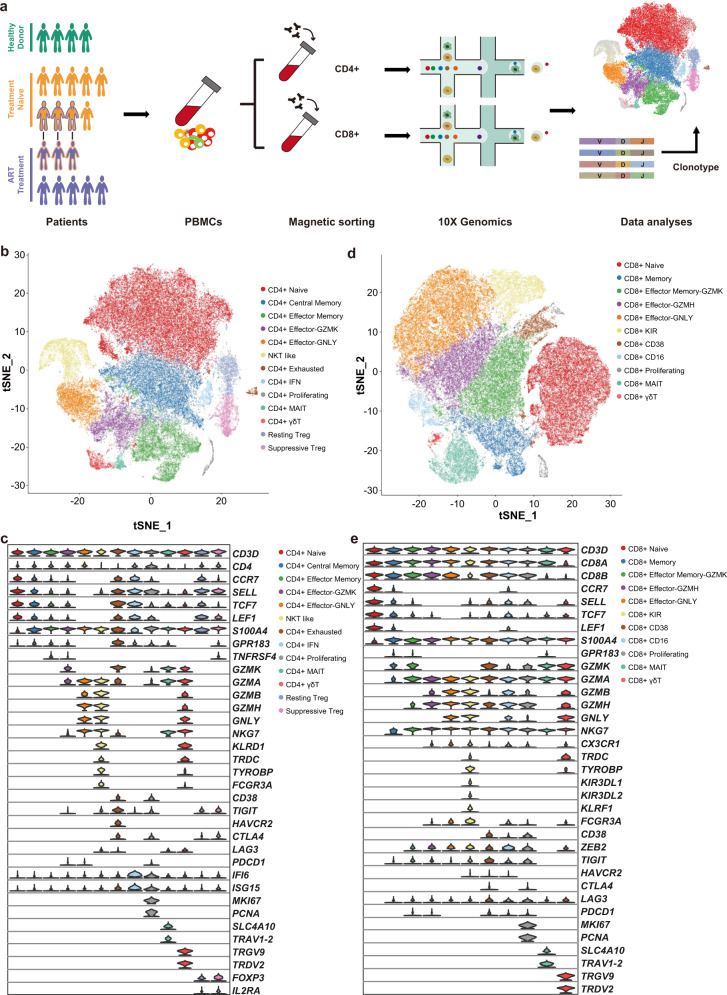
Study design and single-cell transcriptional profiling of CD4^+^ and CD8^+^ T cells from HDs and chronic HIV-1-infected individuals. **a** Schematic showing the overall study design. scRNA-seq was applied to CD4^+^ and CD8^+^ T cells across the three conditions, and the output data were used for TCR profiling and expression analyses. **b** Cellular populations identified for CD4^+^ T cells. The *t*-SNE projection of 79,405 single cells from HD (*n* = 4), TP (*n* = 9), and ART (*n* = 8) samples, showing the formation of 13 clusters with the respective labels. Each dot corresponds to a single cell, colored according to cell types. **c** Violin plots showing the expression distribution of selected canonical cell markers in the 13 clusters of CD4^+^ T cells. Row representing selected marker genes and column representing clusters with the same color in **b**. **d** Cellular populations identified for CD8^+^ T cells. The *t*-SNE projection of 89,155 single cells from HD (*n* = 4), TP (*n* = 9), and ART (*n* = 8) samples, showing the formation of 11 clusters with the respective labels. Each dot corresponds to a single cell, colored according to cell types. **e** Violin plots showing the expression distribution of selected canonical cell markers in the 11 clusters of CD8^+^ T cells. Row representing selected marker genes and column representing clusters with the same color in **d**.

Droplet-based scRNA-seq (10× Genomics) and TCR sequencing were performed on CD4^+^ and CD8^+^ T cells purified from the PBMCs of 14 individuals with chronic HIV-1 infection, with healthy donors (HD; *n* = 4) as controls. These 14 individuals included nine treatment-naive (TP) and 8 with ART (of whom 3 were paired with TP cases) (Fig. 1a and Supplementary Table S1). In total, we obtained transcriptional profiles from 79,405 CD4^+^ and 89,155 CD8^+^ T cells, respectively. Dimensional reduction analysis (*t*-stochastic neighbor embedding [*t*-SNE]) was applied to the expression data and identified 13 CD4^+^ T cell subsets and 11 CD8^+^ T cell subsets according to the expression of canonical gene markers independent of individuals’ origins, read depths, or mitochondrial read counts (Fig. 1b–e and Supplementary Fig. S1a, b, d, e).

As expected, the major subsets of CD4^+^ T cells were naive cells (CD4^+^ Naive: *CCR7*^+^*SELL*^+^) and memory cells (CD4^+^ Central Memory: *S100A4*^+^*GPR183*^+^; CD4^+^ Effector Memory: *S100A4*^+^*GPR183*^+^*TNFRSF4*^+^). Also observed were proliferating (CD4^+^ Proliferating: *MKI67*^+^*PCNA*^+^), CD4^+^ MAIT (*SLC4A10*^+^*TRAV1-2*^+^), CD4^+^ γδT (*TRGV9*^+^*TRDV2*^+^) and two regulatory T subsets (Resting Treg: *FOXP3*^+^*IL2RA*^+^*CCR7*^+^; Suppressive Treg: *FOXP3*^+^*IL2RA*^+^*CTLA4*^+^) (Fig. 1b, c). Three effector CD4^+^ T cell subsets were identified: CD4^+^ Effector-GNLY cells, characterized by high expression of cytotoxic genes including *NKG7*, *GZMA*/*B*/*H,* and *GNLY*; CD4^+^ Effector-GZMK cells, which express a high level of the *GZMK* gene, but low levels of other cytotoxic genes; and natural killer T (NKT)-like cells with high expression of some typical NK cell genes, such as *KLRD1*, *TRDC*, and *FCGR3A* (Fig. 1c). Notably, a subpopulation of CD4^+^ T cells expressed several exhaustion markers and *GZMK* (CD4^+^ Exhausted: *TIGIT*^+^*HAVCR2*^+^*GZMK*^+^); one subpopulation displayed a strong type I interferon (IFN) response (CD4^+^ IFN: *IFI6*^+^*ISG15*^+^). Additionally, CD4^+^ Effector-GNLY and NKT like cells showed high expression of some classic type 1 helper T (Th1) cell genes, such as *TBX21*, *CCL5,* and *GZMB*, implying that they were Th1-like cells. The expression of *GATA3* and *IL4*, two classic type 2 helper T (Th2) cells genes, were enriched in CD4^+^ Effector-GZMK and CD4^+^ Effector Memory cells (Supplementary Fig. S1c).

The major CD8^+^ T cell subpopulations were effector cells, with the exception of typical naive (CD8^+^ Naive: *CCR7*^+^*SELL*^+^), memory (CD8^+^ Memory: *S100A4*^+^*GPR183*^+^), proliferating (CD8^+^ Proliferating: *MKI67*^+^*PCNA*^+^), CD8^+^ MAIT (*SLC4A10*^+^*TRAV1-2*^+^) and CD8^+^ γδT cells (*TRGV9*^+^*TRDV2*^+^). Similarly, the CD8^+^ Effector-GNLY cells were characterized by high expression of the *GNLY* gene, and the CD8^+^ Effector Memory-GZMK cells displayed relatively high expression levels of the *GZMK* gene (Fig. 1e). However, the CD8^+^ Effector-GZMH cells did not express the *GZMK* and *GNLY* genes. The CD8^+^ killer cell immunoglobulin-like receptor (KIR) cells showed a similar expression pattern to the CD8^+^ Effector-GNLY cells (Fig. 1e). In addition, two effector CD8^+^ T cells were defined (CD8^+^ CD38 and CD8^+^ CD16), highly expressing exhaustion markers (*TIGIT* and *LAG3*). Interestingly, the CD8^+^ CD38 cells showed high *CD38* and *GZMK* expression but no *GNLY* expression; the CD8^+^ CD16 cells expressed high *FCGR3A* and *GNLY* but low *GZMK* (Fig. 1e). Thus, we clearly defined the composition of T cell subpopulations in the peripheral blood of individuals with chronic HIV-1 infection.

### Transcriptomic changes of CD4^+^ T cell subsets across disease conditions

**Fig. 2:**
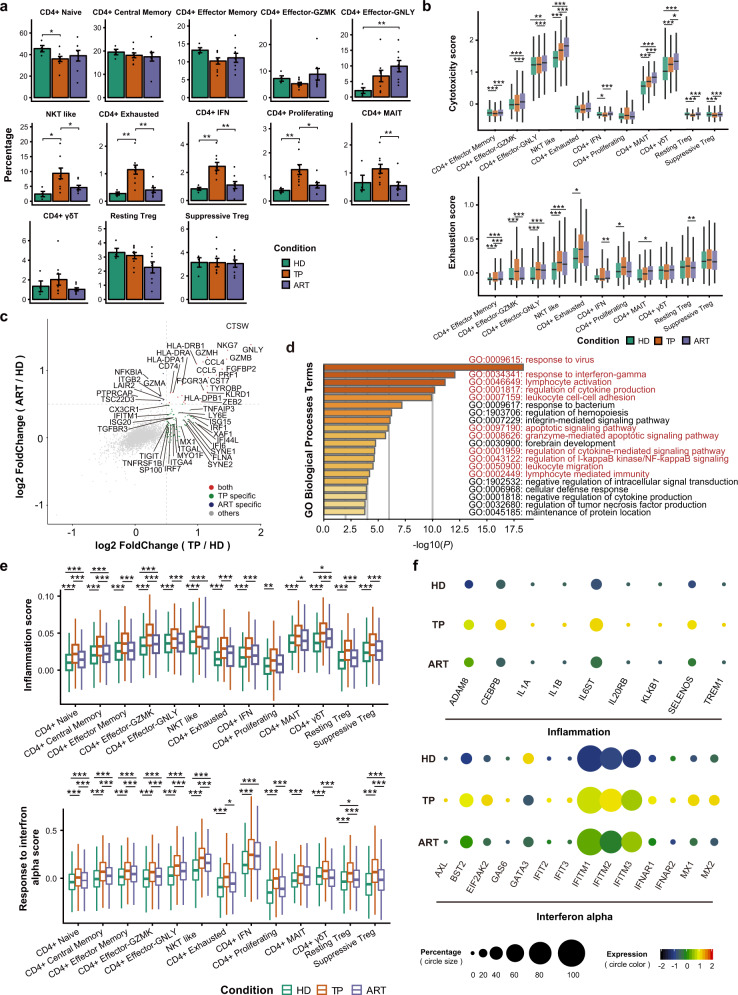
Immunological features of CD4^+^ T subsets across the three conditions. **a** Condition preference of each cluster. *Y*-axis: average percent of samples across the three conditions. Conditions are shown in different colors. Each bar plot represents one cell cluster. Error bars represent ±S.E.M. for 4 healthy donors and 14 chronic HIV-1-infected individuals. *P < 0.05; **P < 0.01; two-sided unpaired Mann–Whitney *U*-test. **b** Box plots of the cytotoxicity and exhaustion scores across different clusters and conditions. Conditions are shown in different colors. Horizontal lines represent median values, with whiskers extending to the farthest data point within a maximum of 1.5× interquartile range. *P < 0.01; **P < 0.001; ***P < 0.0001; two-sided unpaired Dunn’s (Bonferroni) test. **c** DEGs of CD4^+^ T cells from the TPs or ARTs in comparison with those from the HDs. Each red dot denotes an individual gene with Benjamini–Hochberg adjusted *P* value (two-sided unpaired Mann–Whitney *U*-test) ≤0.01 and average log_2_(fold change) ≥0.5 for the TP/HD and ART/HD comparisons. Example genes are labeled with the gene name. **d** Gene enrichment analyses of the DEGs in TPs compared with healthy donors. GO terms are labeled with name and id and sorted by −log10 (*P*) value. A darker color indicates a smaller *P* value. The top 20 enriched GO terms are shown. Interesting terms are labeled in red color. **e** Similar to **b** but for inflammation and IFN-α response score. **f** Dot plot showing expression of some genes associated with inflammation and IFN-α response processes across the three conditions. The size of the circle indicates the percentage of cells expressing pathway-associated genes under each condition. The color of the circle represents the expression levels of pathway-associated genes under each condition, and the color red means a relatively high expression level, and black means a relatively low expression level.

We then evaluated the distribution of each cluster across the three conditions, i.e., TP, ART, and HD (Fig. 2a). Globally, there were reduced proportions of naive-state T cell subsets, particularly CD4^+^ naive T cells in TPs, which could not return to the normal levels detected in the HDs even after treatment. The proportions of NKT-like, exhausted, IFN response, proliferating CD4^+^ T and CD4^+^ MAIT cells peaked in the TPs and decreased in the ARTs. Of particular interest, the percentage of the CD4^+^ Effector-GNLY subset was further increased after ART.

The cytotoxicity and exhaustion state of effector-state T cell subsets across the three conditions were scored to investigate the functional state of CD4^+^ T cells. CD4^+^ Effector-GNLY, NKT-like, CD4^+^ MAIT, and CD4^+^ γδT cells showed higher cytotoxicity than cells in other subsets (Fig. 2b). These highly cytotoxic cells from the ARTs exhibited a higher cytotoxic state than those of the TPs (Fig. 2b and Supplementary Fig. S2a), suggesting that the toxicity of effector cells accumulates after HIV-1 infection and the immune system was persistently activated as opposed to returning to a homeostatic state after ART. Nearly all CD4^+^ T cell subsets in the TPs exhibited an exhausted state (Fig. 2b and Supplementary Fig. S2a), which agrees with a previous study showing that CD4^+^ T cells from individuals with chronic HIV-1 infection were highly exhausted and functionally impaired^29^. Following ART, the exhaustion state returned to nearly normal levels observed in the HDs in all T cell subsets except for three highly cytotoxic subsets (CD4^+^ Effector-GNLY, NKT-like, and CD4^+^ MAIT). These results emphasize the notion that dysfunctional CD4^+^ T cells persist even after successful ART^30^.

The transcriptomic profiles of CD4^+^ T cells in effector-state (including CD4^+^ Effector Memory, CD4^+^ Effector-GZMK, CD4^+^ Effector-GNLY, NKT-like, CD4^+^ IFN, and CD4^+^ Exhausted clusters) were compared between the TP or ART and HD conditions. Cells from the TPs and ARTs showed higher expression of T cell cytotoxicity-associated genes and major histocompatibility complex class II (MHC-II) genes, whereas TPs had more differentially expressed genes (DEGs) related to antiviral responses (Fig. 2c). The DEGs upregulated in chronic HIV-1-infected individuals were involved in processes that included IFN responses, lymphocyte activation, cytokine production, cell killing, leukocyte cell-cell adhesion/migration, apoptotic signaling pathways, and inflammatory responses (Fig. 2c, d and Supplementary Fig. S2b).

We further compared the transcriptional changes of each CD4^+^ cell subset between TP and ART conditions. For all CD4^+^ T cell subsets, we found that limited genes were significantly upregulated, while more genes were significantly downregulated after ART (Supplementary Fig. S2c). These downregulated DEGs were involved in defense response to the virus, IFN responses, cytokine production, and the apoptotic signaling pathway (Supplementary Fig. S2d). In line with the DEG enrichment results, the scoring system showed that chronic HIV-1 infection induced upregulation of some essential processes, including response to IFN-α, inflammatory responses, apoptosis, and migration in all CD4^+^ T cell subsets (Fig. 2e, f and Supplementary Fig. S2e, f).

### Clonal expansion in CD4^+^ Effector-GNLY cells in individuals with chronic HIV-1 infection

**Fig. 3:**
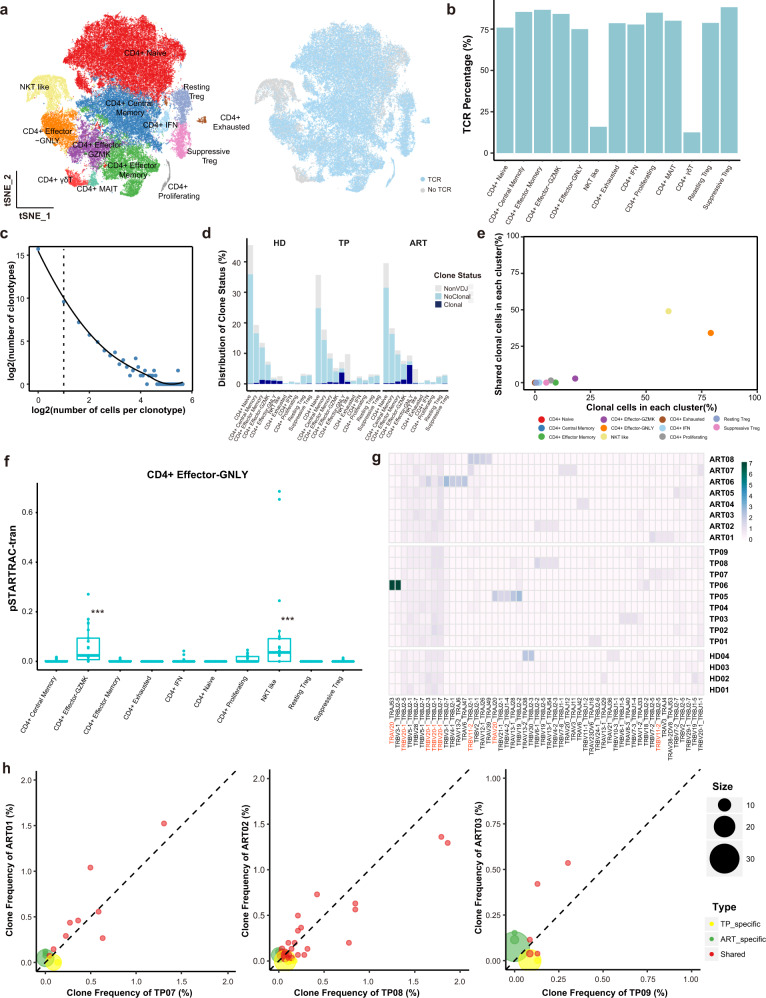
Clonal expansion of CD4^+^ T cells and clonal maintenance of CD4^+^ Effector-GNLY cells following ART. **a** *t*-SNE of CD4^+^ T cells. Clusters are denoted by color labeled with inferred cell types (left) and TCR detection (right). Cells are colored the same in (Fig. 1b). **b** Bar plots showing the percent of TCR detection in each CD4^+^ T cell cluster. **c** The association between the number of CD4^+^ T cell clones and the number of cells per clonotype. The dashed line separates non-clonal and clonal cells, with the latter identified by repeated usage of TCRs. LOESS fitting is labeled as the solid line showing a negative correlation between the two axes. **d** The distribution of the clone state of CD4^+^ T cells in each cluster across the three conditions. **e** Comparison between the fraction of clonal cells in each subset (*X*-axis) and percentage of cells with TCRs shared across clusters (*Y*-axis). **f** Developmental transition of CD4^+^ Effector-GNLY cells with other CD4^+^ cells quantified by pairwise STARTRAC-tran indices for each patient (*n* = 14). ***P < 0.001, Permutation test. **g** Top 5 paired V-J usage of TRA/B genes across four healthy donors and 14 chronic HIV-1-infected individuals. **h** Scatterplots comparing TCR clone frequencies between pre- and post-ART treatment for three participants. The size of the dot corresponds to the number of distinct clonotypes. The clonotypes that are shared between pre- and post-ART are displayed in blue, and those only in TP or ART are in green or orange, respectively.

Subsequently, to gain insight into the clonal relationship among individual CD4^+^ T cells, the TCR sequences were reconstructed (Supplementary Table S2). Briefly, >75% of cells in all subsets had matched TCR information except for the NKT-like and CD4^+^ γδT subsets (Fig. 3a, b). There were different degrees of clonal expansion among the CD4^+^ T cell subsets, and effector cells displayed higher clonal expansions than naive or memory cells (Fig. 3c, d). Moreover, CD4^+^ Effector-GNLY cells from the TPs and ARTs showed more apparent clonal expansion than other effector cells (Fig. 3d). In conjunction with the above findings, these results suggest that CD4^+^ Effector-GNLY cells were overactivated during chronic HIV-1 infection. CD4^+^ Effector-GNLY cells and NKT-like cells contained high proportions of inter-cluster clonal cells (Fig. 3e). CD4^+^ Effector-GNLY cells shared numerous clones with CD4^+^ Effector-GZMK cells and NKT-like cells, suggesting that effector CD4^+^ T cells underwent dynamic state transitions (Fig. 3e, f).

We analyzed the usage of V(D)J genes among individuals with chronic HIV-1 infection and HDs and investigated the gene preference of TCRs in CD4^+^ T cells across the three conditions. Heterogeneous usage of paired V-J genes was observed among different individuals; however, some commonly used paired V-J genes were observed among individuals with chronic HIV-1 infection and HDs (Fig. 3g). Additionally, half of the widely used paired V-J genes shared *TRBV20-1* (Fig. 3g and Supplementary Fig. S3). HIV-1 specific paired V-J genes were not found, but the usage of *TRAV20* was upregulated in two TPs (TP05 and TP06), and *TRBV11-2* was enriched in one TP (TP07) and three ARTs (ART01, ART07, and ART08) (Fig. 3g).

Further investigation into the fate of clonal CD4^+^ T cell subsets after ART was undertaken. Unlike in naive and memory CD4^+^ T cells, obvious clonal maintenance in effector CD4^+^ T cells was revealed (Fig. 3h and Supplementary Fig. S4a–c). Almost all large pre-treatment clones (clone size >5) were retained after ART (Fig. 3h and Supplementary Fig. S4b); these persistently expanded clones belonged to CD4^+^ Effector-GNLY cells (Supplementary Fig. S4c). These results suggest that CD4^+^ Effector-GNLY cells may represent the dominant clonal CD4^+^ T cells in individuals with a chronic HIV-1 infection or after ART.

To track the transcriptional changes of the same clonotypes, we evaluated the DEGs of cells belonging to the same TCR but showing different trends of variations in the clonal size after treatment (stable clones: clonal size is stable after ART; contracting clones: clonal size contracts after ART; expanding clones: clonal size continues to expand after ART) before and after ART. Universally, ART reduced the expression of interferon response associated genes (e.g., *IRF1* and *IFI6*) in all clones in CD4^+^ cells. The *CXCR4* is another gene whose expression level was descending in all clones after ART (Supplementary Fig. S4d, e).

### Characterization of CD8^+^ T cell subsets in untreated and treated individuals with HIV-1 infection

**Fig. 4:**
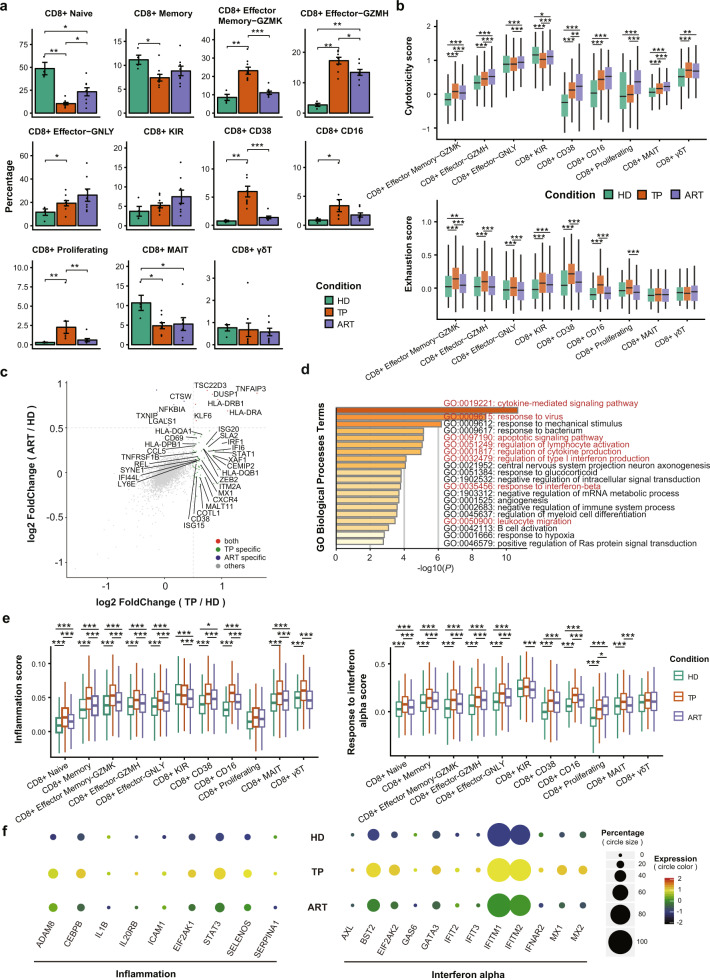
Characterizations of CD8^+^ T cell subsets across the three conditions. **a** Condition preference of each cluster. *Y*-axis: average percent of samples across the three conditions. Conditions are shown in different colors. Each bar plot represents one cell cluster. Error bars represent ±S.E.M. for four healthy donors and 14 chronic HIV-1-infected individuals. *P < 0.05; **P < 0.01; ***P < 0.001; two-sided unpaired Mann–Whitney *U*-test. **b** Box plots of the cytotoxicity and exhaustion scores across different clusters and conditions. Conditions are shown in different colors. Horizontal lines represent median values, with whiskers extending to the farthest data point within a maximum of 1.5× interquartile range. *P < 0.01; **P < 0.001; ***P < 0.0001; two-sided unpaired Dunn’s (Bonferroni) test. **c** DEGs of CD8^+^ T cells from the TPs or ARTs in comparison with those from the HDs. Each red dot denotes an individual gene with Benjamini–Hochberg adjusted *P* value (two-sided unpaired Mann–Whitney *U*-test) ≤0.01 and average log_2_(fold change) ≥0.5 for the TP/HD and ART/HD comparisons. Example genes are labeled with the gene name. **d** Gene enrichment analyses of the DEGs in TPs compared with healthy donors. GO terms are labeled with name and id and sorted by −log10 (*P*) value. A darker color indicates a smaller *P* value. The top 20 enriched GO terms are shown. Interesting terms are labeled in red color. **e** Similar to **b** but for inflammation and IFN-α response score. **f** Dot plot showing expression of some genes associated with inflammation and IFN-α response processes across the three conditions. The size of the circle indicates the percentage of cells expressing pathway-associated genes under each condition. The color of the circle represents the expression levels of pathway-associated genes under each condition, and the color red means a relatively high expression level, and black means a relatively low expression level.

Integrated analysis of the composition of CD8^+^ T cells across the three conditions revealed dramatic alterations in the CD8^+^ T cell subsets. Notably, HIV-1 infection induced a sharp decrease in proportions of CD8^+^ naive, memory, and MAIT cells. Their proportions increased slightly but remained at comparatively lower levels even after ART compared to HDs (Fig. 4a). Furthermore, the pools of CD8^+^ Effector Memory-GZMK, CD8^+^ Effector-GZMH, CD8^+^ CD38, CD8^+^ CD16, and CD8^+^ proliferating T cells were enlarged in TPs and then decreased after ART. As with the CD4^+^ Effector-GNLY cells, it is worth noting that the percentage of CD8^+^ Effector-GNLY cells was also increased after ART.

Almost all CD8^+^ T cell subsets from the ARTs exhibited a higher or comparable cytotoxic state to those from the TPs, except CD8^+^ Effector Memory-GZMK cells whose cytotoxic state was significantly lower than those in TPs (Fig. 4b and Supplementary Fig. S5a). During chronic HIV-1 infection, most CD8^+^ T cell subsets exhibited strong exhaustion signatures; this exhaustion state could be partially eased after ART (Fig. 4b and Supplementary Fig. S5a). This result indicates that effector CD8^+^ T cells in individuals with chronic HIV-1 infection exhibited exhausted and dysfunctional status.

The differences in the expression patterns of six effector CD8^+^ cells (CD8^+^ Effector Memory-GZMK, CD8^+^ Effector-GNLY, CD8^+^ Effector-GZMH, CD8^+^ KIR, CD8^+^ CD38, and CD8^+^ CD16 cells) were investigated across the three conditions. Generally, most DEGs were observed in the TP condition compared to HD (Fig. 4c). The significantly upregulated pathways were involved in IFN responses, apoptotic signaling, lymphocyte activation, cytokine production, and leukocyte migration (Fig. 4c, d and Supplementary Fig. S5b).

To investigate the transcriptomic changes of each CD8^+^ cell subset between TP and ART conditions, we first calculated the differential expression genes of these subsets. Similar to the CD4^+^ T subsets, DEGs associated with IFN responses, cytokine production, response to the virus, and the apoptotic signaling pathway were significantly downregulated in individuals after ART (Supplementary Fig. S5c, d), suggesting a consistent response by adaptive immune cells to ART. In addition, some DEGs (e.g., *IL7R*, *GZMB*, and *GNLY*) that are closely related to adaptive immune response were upregulated after ART, especially in CD8^+^ CD38, CD8^+^ Proliferating, and CD8^+^ Effector-GNLY subsets (Supplementary Fig. S5d). Moreover, almost all CD8^+^ T cell subsets in the TPs showed higher activation scores of several processes, such as inflammatory responses, response to IFN-α, apoptosis, and migration (Supplementary Fig. S5e, f).

### Expanded Effector CD8^+^ T cells persisted after ART

**Fig. 5:**
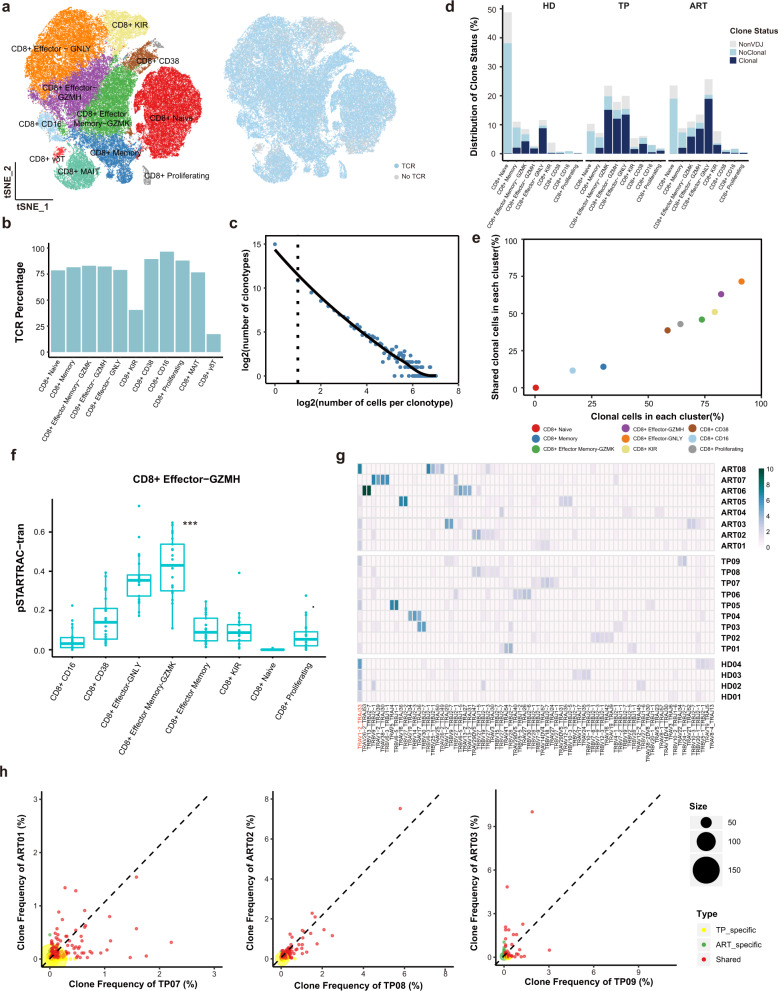
Clonal expansion of CD8^+^ T cells and clonal maintenance of CD8^+^ Effector-GNLY cells following ART. **a** *t*-SNE of CD8^+^ T cells. Clusters are denoted by color labeled with inferred cell types (left) and TCR detection (right). Cells are colored the same as in Fig. 1d. **b** Bar plots showing the percent of TCR detection in each CD8+ T cell cluster. **c** The association between the number of CD8^+^ T cell clones and the number of cells per clonotype. The dashed line separates non-clonal and clonal cells, with the latter identified by repeated usage of TCRs. LOESS fitting is labeled as the solid line showing a negative correlation between the two axes. **d** The distribution of the clone state of CD8^+^ T cells in each cluster across the three conditions. **e** Comparison between the fraction of clonal cells in each subset (*X*-axis) and percentage of cells with TCRs shared across clusters (*Y*-axis). **f** Developmental transition of CD8^+^ Effector-GZMH cells with other CD8^+^ cells quantified by pairwise STARTRAC-tran indices for each patient (*n* = 14). ***P < 0.001, Permutation test. **g** Top 5 paired V-J usage of TRA/B genes across four healthy donors and 14 chronic HIV-1-infected individuals. **h** Scatterplots comparing TCR clone frequencies between pre- and post-ART treatment for three participants. The size of the dot corresponds to the number of distinct clonotypes. The clonotypes that are shared between pre- and post-ART are displayed in blue, and those only in TP or ART are in green or orange, respectively.

TCR analysis revealed that the percentage of detected TCRs was >75% in each subset except the CD8^+^ KIR and CD8^+^ γδT cells (Fig. 5a, b). The reconstructed TCR sequences are listed in Supplementary Table S3. Compared with CD4^+^ T cells, there was more obvious clonal expansion in the CD8^+^ T cell subsets, with the exception of naive or memory cells, which displayed similarly negligible clonal expansion (Fig. 5c, d). The effector CD8^+^ T cell subsets (CD8^+^ Effector-GZMH, CD8^+^ Effector Memory-GZMK, CD8^+^ Effector-GNLY, CD8^+^ KIR, and CD8^+^ CD38) not only displayed high proportions of clonal cells (Fig. 5d), but also contained high proportions of inter-cluster clonal cells (Fig. 5e, f), suggesting that CD8^+^ T cells underwent extensive expansion and state transitions (Fig. 5a, f).

The usage of V(D)J genes across the three conditions was compared to explore how HIV-1 infection and ART influence the process of V(D)J rearrangements in CD8^+^ T cells. In contrast to CD4^+^ T cells, the CD8^+^ T cells exhibited more significant heterogeneity in using paired V-J genes among different individuals (Fig. 5g). The V-J gene pair *TRAV1-2*/*TRAJ33*, the only commonly used paired V-J gene in the HDs, was decreased after HIV-1 infection (Fig. 5g and Supplementary Fig. S6). The usage of *TRBV27* was widely upregulated in five TPs (TP01, TP06, TP07, TP08, and TP09) and six ARTs (ART02, ART04, ART05, ART06, ART07, and ART08) (Fig. 5g); however, five of them (TP01, TP09, ART05, ART06, and ART07) shared the same HLA-A02 type and two of them (TP08 and ART02) had HLA-A11 type (Supplementary Table S4). This result suggests that HLA type might affect the preferential usage of V(D)J genes in activated CD8^+^ T cells during chronic HIV-1 infection.

Next, we traced the clonal CD8^+^ T cell fates after ART. Several clonal cells were retained in the effector CD8^+^ T cells even after ART (Fig. 5h and Supplementary Fig. S7a, b). Interestingly, most clonal cells in the CD8^+^ Effector-GZMH, CD8^+^ Effector Memory-GZMK, and CD8^+^ CD38 subsets were more likely to decrease after treatment; however, some clones in the CD8^+^ Effector-GNLY subset were retained or increased (Supplementary Fig. S7c). Collectively, the increased proportions and clonal expansion of the CD8^+^ Effector-GNLY cells suggest that these activated and expanded CD8^+^ cells persist in individuals with chronic HIV-1 infection even after treatment, which might support the notion that the activation and expansion of CD8^+^ T cells are driven by nonspecific bystander activation^31^.

We also investigated the influence of ART on CD8^+^ T cells sharing the same clonotype. We found that ART reduced the expression of interferon response associated genes (e.g., *IRF1* and *IFI6*) and MHC-II genes in all clones in CD8^+^ cells (Supplementary Fig. S7d, e). Apparently, the contracting clones displayed a lower expression level of *TPT1* before ART (Supplementary Fig. S7d, e).

### Implications of T cell compositions in individuals with chronic HIV-1 infection

**Fig. 6:**
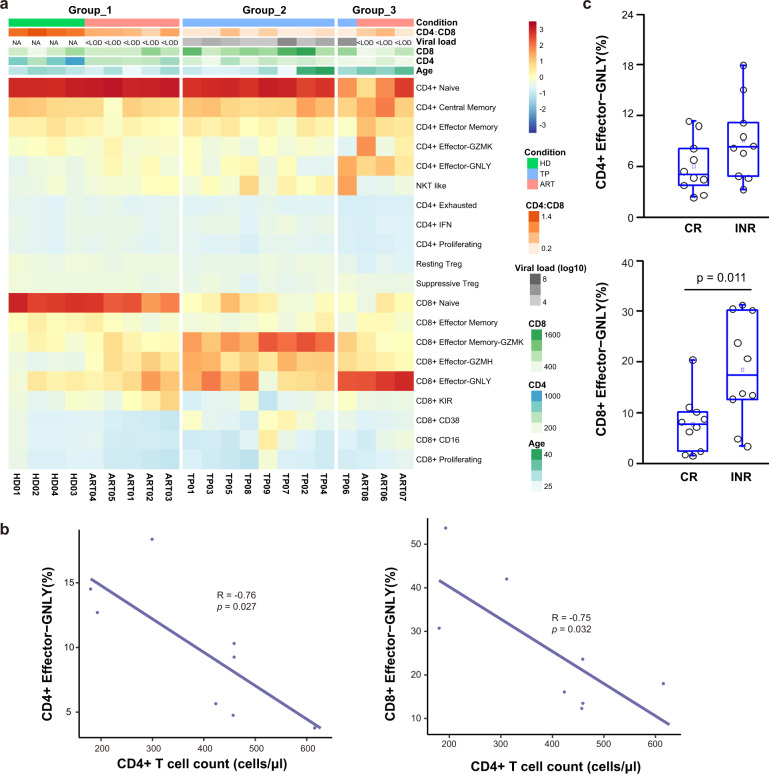
Clinical implications of GNLY^+^ effector T cells in chronic HIV-1-infected individuals. **a** Hierarchical clustering of compositions of CD4^+^ and CD8^+^ T cells in each patient. Top heat map showing clinical information (condition, viral load, CD4/CD8, CD8 count, CD4 count, CD3 count, and age) of 4 healthy donors and 14 chronic HIV-1-infected individuals. **b** Relationship between the proportions of CD4^+^ Effector-GNLY and CD8^+^ Effector-GNLY and the numbers of CD4^+^ T cells in PBMCs of ARTs. **c** Box plots showing frequencies of CD4^+^ Effector-GNLY (top) and CD8^+^ Effector-GNLY (bottom) in clinical CRs (*n* = 10) and INRs (*n* = 10). All differences with *P* < 0.05 are indicated; two-sided unpaired Mann–Whitney *U*-test.

Hierarchical clustering based on T cell compositions in the HDs and individuals with chronic HIV-1 infection yielded three groups (Fig. 6a). Group_1 consisted of all HDs and five ARTs, with features such as abundant naive CD4^+^ and CD8^+^ cells. Group_2 only included TPs with reduced naive CD4^+^ cells, significantly low naive CD8^+^ cells, and increased effector CD8^+^ cells. Notably, Group_3 appeared complicated and included two INRs (ART07 and ART08), as well as ART06 and TP06, who all had minimal naive CD4^+^ and CD8^+^ cells, but maximally expanded CD4^+^ and CD8^+^ Effector-GNLY cells (Fig. 6a and Supplementary Fig. S8a, b).

During ART, the peripheral CD4^+^ T cell counts in individuals with chronic HIV-1 infection represent an important immune parameter for host immune restoration. Here, we found that naive CD4^+^ and CD8^+^ cells correlated positively with CD4^+^ T cell counts. However, the CD4^+^ and CD8^+^ Effector-GNLY subsets correlated negatively with CD4^+^ T cell counts in the ARTs (Fig. 6b and Supplementary Fig. S8c).

To investigate the features of CD4^+^ and CD8^+^ Effector-GNLY cells, we gated these cells through flow cytometry based on the defined markers in our scRNA-seq data (Fig. 1c, d and Supplementary Fig. S8d). Both our scRNA-seq data and FACS data showed that the percentage of cells highly expressing cytotoxic genes (such as *GZMB* and *CX3CR1*) and differentiation-associated transcription factors (*T-bet* and *EOMES*) in CD4^+^ or CD8^+^ Effector-GNLY subsets was much higher than that in all CD4^+^ or CD8^+^ cells (Supplementary Fig. S9a–d), which demonstrated the differentiated and cytotoxic activities of both CD4^+^ and CD8^+^ Effector-GNLY cells. Besides, we also observed that cells highly expressing *PDCD1* were greatly enriched in CD4^+^ Effector-GNLY cells (Supplementary Fig. S9a, c).

To examine whether HIV-1-infected individuals with low CD4^+^ T cell counts have enrichment of these two GNLY^+^ effector T cells, we further evaluated the relative percentage of CD4^+^ and CD8^+^ Effector-GNLY cells in an additional cohort comprising 10 CRs and 10 INRs (Supplementary Table S5). We found that CD8^+^ Effector-GNLY cells were significantly enriched in the INRs (Fig. 6c and Supplementary Fig. S8d). Our data indicate that enrichment of CD8^+^ GNLY^+^ effector T cells may represent poor immune restoration in some ARTs and TPs, although their exact biological functions warrant future studies.

## Discussion

HIV-1 chronically infects host CD4^+^ T lymphocytes and further affects a wide variety of immune cells, including CD8^+^ T lymphocytes, which serve as a central restriction factor for viral replication^32,33^. Previous studies have demonstrated some immune characteristics and changes of T cells in chronic HIV-1-infected individuals^3,4,9,11^. However, an integrated view of the global T cell subsets is still lacking, which makes it difficult to achieve a better understanding of the dynamic pathogenesis and its relation to immune restoration and outcome. Here, we present the transcriptional landscapes combined with the TCR repertoires of total T cells at single-cell resolution in chronic HIV-1-infected during the natural course of the disease and under ART.

Our study reveals abnormal global changes in the CD4^+^ and CD8^+^ T cells in the TPs, including a significant loss of naive CD4^+^ and CD8^+^ T cell subsets yet a profound increase in CD4^+^ and CD8^+^ T cell subsets with high cytotoxicity and clonal expansion or proliferating features. Due to the cytotoxicity roles and clonal expansion of CD8^+^ T cells, the loss of the naive CD8^+^ subset was more obvious compared with that of the naive CD4^+^ subset upon HIV-1 infection. Additionally, the proportions of three effector CD8^+^ T cell subsets, namely CD8^+^ Effector Memory-GZMK, CD8^+^ Effector-GNLY, and CD8^+^ Effector-GZMH were dramatically increased in the TPs. These three effectors responded differently to ART. ART induced a near entire eclipse of CD8^+^ Effector Memory-GZMK cells, but only partially influenced CD8^+^ Effector-GZMH cells. Interestingly, the increased CD8^+^ Effector-GNLY cells were further expanded during ART, the same to the CXCR5^+^CD8^+^ T subset cells who underwent proliferative burst after PD-1 blockade^34^. Whether there is any relationship between our CD8^+^ Effector-GZMY cells and the CXCR5^+^CD8^+^ T subset cells remains to be further verified. Simultaneously, a further increase of CD4^+^ Effector-GNLY cells was also observed. The different cytotoxic molecular expression profiles among the three CD8^+^ effector T cells could be associated with various regulations during or after transcription. How these three effector CD8^+^ T cell subsets play synergistic roles requires further studies on chronic HIV-1 infection.

Type I IFNs and other inflammatory cytokines play essential antiviral roles in acute and hyperacute HIV-1 infection; however, they are likely detrimental in chronic HIV-1 infection^35^. Here, IFN-α response was significantly increased in all T cell subsets of the TPs and was reduced in the majority of the ARTs; however, IFN-α response was nevertheless higher than that in the HDs. Studies on humanized mice have shown that ART in combination with blocking of type I IFN signaling successfully suppressed HIV-1 replication, reversed immune dysfunction, and reduced the HIV-1 reservoir in vivo^36,37^. These observations imply the necessity of regulating the type I IFN pathway in the ARTs with immune therapy. Here, we observed persistent inflammation, immune overactivation, increased apoptosis, and migration of T cell subsets in individuals with chronic HIV-1 infection; these changes could be partially suppressed by ART but did not return to steady states as in the HDs. These abnormalities have been considered potential mediators that closely correlate with poor immune restoration and non-AIDS-related morbidity and mortality in the ARTs^12^. Our data link the abnormal responses of T cell subsets with clinical outcomes in the ARTs, but the underlying mechanisms need future studies to elucidate.

In the clinical setting, there is no consensus or unified criteria of INRs for the ARTs. The ARTs in Group_3 exhibited different features from the HDs and other ARTs with better clinical outcomes, including the fewest naive CD4^+^ and CD8^+^ cells but maximally expanded CD4^+^ and CD8^+^ Effector-GNLY cells in the peripheral blood. Two of four were identified as INRs, with poor immune restoration. Interestingly, the only one TP in Group_3, TP06, could not achieve a better CD4^+^ T cell count even after 1-year treatment (Supplementary Fig. S9e). Whether this immune evidence in Group_3 can be used for predicting INR warrants further studies. Our data also indicate that early initiation of ART would favor the efficient restoration of T cell composition, as shown in ART01, ART02, and ART03, which further confirms the premise that starting ART in individuals with high CD4 count likely provides the best opportunity for immune restoration^14^.

Microbial translocation has been regarded as a crucial event for determining the degree of immune restoration after ART, and the levels of microbial translocation remain elevated two-fold compared to that of HDs^38,39^. In addition, the microbial infection can induce *GNLY* expression in CD4^+^ and CD8^+^ T cells^40^. In our study, two INRs displayed excessive expansion of both CD4^+^ and CD8^+^ Effector-GNLY cells. Thus, it is necessary to further study whether increased CD4^+^ and CD8^+^ Effector-GNLY cells are associated with microbial translocation and microbiota disturbance due to gut mucosal injury induced by chronic HIV-1 infection.

The present study has several limitations. First, we did not analyze other immune cells, particularly the B lymphocytes, NK cells, and DCs. Second, lymph nodes are an important site for HIV-1 replication^41^; therefore, further investigation would ideally include immune cell populations from lymph nodes. Third, our sample size was limited, e.g., there were only three individuals with matched pre- and post-ART longitudinal samples; enrolling more TPs, INRs and CRs may aid the definition of the fundamental event related to immune restoration.

Overall, we used scRNA-seq to portray the global landscape of peripheral T cells and the immune responses in chronic HIV-1-infected individuals. Our findings systematically illustrate the marked changes in T cell composition, molecular pathways and functional features in individuals with chronic HIV-1 infection with or without ART. Our study may point out new research directions for a deeper understanding of the pathogenesis of HIV infection and also supply evidence to develop novel targeted immune therapies for the disease.

## Materials and methods

### Patient enrollment and clinical characteristics

We enrolled nine TP individuals with chronic HIV-1 infection prepared to receive ART after their 1-week diagnosis, and eight individuals who had undergone successful ART for >6 months with undetectable plasma HIV-1 RNA after least two consecutive tests (three were paired with the TP cases, Fig. 1a). The diagnostic criteria were defined as in the previous reports^9,12,42^. The exclusion criteria included co-infection with hepatitis B virus or hepatitis C virus, tuberculosis, pregnancy, and other opportunistic infections^9,41^. CRs were defined as individuals who have received successful ART for ≥2 years with peripheral CD4^+^ T cell count above 350 cells/µL and plasma HIV-1 RNA at under-detectable level, and INRs were defined as individuals who have received successful ART for ≥2 years with peripheral CD4^+^ T cell count below 200 cells/µL and plasma HIV-1 RNA at under-detectable level^43–45^. The participants’ detailed characteristics and ART regimens are listed in Table S1. This study was approved by the Ethics Committee of the Fifth Medical Center of PLA General Hospital with project number 2016-201-D. Written informed consent was obtained from each participant.

### Preparation of single-cell suspensions

PBMCs were initially isolated by Ficoll-Hypaque density gradient centrifugation from EDTA-anticoagulated blood on the admission of 14 individuals with chronic HIV-1 infection and 4 HDs. CD4^+^ and CD8^+^ T cells were immediately purified from fresh PBMCs using CD4 negative magnetic selection (130-045-101, Miltenyi Biotech) and CD8 positive magnetic selection (130-045-201, Miltenyi Biotech) isolation kits, respectively. All cell separations were performed according to the manufacturer’s instructions. For each isolated CD4^+^ and CD8^+^ cells both had >97% purity and >90% viability.

### Droplet-based single-cell sequencing

Using a Single Cell 5′ Library & Gel Bead Kit (10× Genomics) and Chromium Single Cell A Chip Kit (10× Genomics), cell suspensions (300–600 living cells/µL as determined by Countstar) were loaded onto a Chromium Single-cell controller (10× Genomics) to generate single-cell gel beads in emulsion (GEMs) according to the manufacturer’s protocol. Briefly, single cells were suspended in phosphate-buffered saline (PBS) containing 0.04% bovine serum albumin (BSA). The cells were added to each channel, and approximately 50% of input cells were recovered. The captured cells were lysed, and the released RNA was barcoded through reverse transcription in individual GEMs. Reverse transcription was performed on a S1000™ Thermal Cycler (Bio-Rad Laboratories, Hercules, CA) at 53 °C for 45 min, followed by 85 °C for 5 min, and held at 4 °C. Complementary DNA (cDNA) was generated and amplified, and its quality was assessed using an Agilent 4200 system (performed by CapitalBio Technology, Beijing, China). Following the manufacturer’s introductions, scRNA-seq libraries were constructed using a Single Cell 5′ Library & Gel Bead Kit, Single Cell V(D)J Enrichment Kit, and Human T Cell (1000005). The libraries were sequenced using an Illumina NovaSeq 6000 sequencer with a paired-end 150 bp (PE150) reading strategy (performed by CapitalBio Technology, Beijing, China).

### scRNA-seq data processing

Raw gene expression matrices were generated for each sample by the Cell Ranger (v.3.0.2) Pipeline coupled with human reference version GRCh38. The output filtered gene expression matrices were analyzed by R software (v.3.5.3) with the Seurat^46^ package (v.3.0.0). In brief, genes expressed at a proportion of >0.1% of the data, and cells with >200 genes detected were selected for further analysis. Low-quality cells were removed if they met the following criteria: (1) <1000 unique molecular identifiers (UMIs); (2) <500 genes; or (3) >10% UMIs derived from the mitochondrial genome. Following the removal of low-quality cells, the gene expression matrices were normalized by the NormalizeData function, and 2000 features with high cell-to-cell variation were calculated using the FindVariableFeatures function. The RunPCA function was conducted with default parameters on linear-transformation scaled data generated by the ScaleData function to reduce the dimensionality of the datasets. Subsequently, the ElbowPlot, DimHeatmap, and JackStrawPlot functions were used to identify the true dimensionality of each dataset, as recommended by the Seurat developers. Finally, cells were clustered using the FindNeighbors and FindClusters functions, and non-linear dimensional reduction was performed with the RunTSNE function with default settings. For CD4^+^ T cells, the resolution was set to 0.5, and the resolution was set to 0.4 for CD8^+^ T cells. All details regarding the Seurat analysis performed in this work are in the website tutorial (https://satijalab.org/seurat/v3.0/pbmc3k_tutorial.html).

### Multiple dataset integration

The integration methods, described at (https://satijalab.org/seurat/v3.0/integration.html), were employed to compare cell types and proportions across the three conditions^47^. The Seurat package (v.3.0.0) was used to assemble multiple distinct scRNA-seq datasets into an integrated and unbatched dataset. In brief, 2000 features were identified with high cell-to-cell variation, as described above. Anchors between individual datasets were identified with the FindIntegrationAnchors function. These anchors were inputted into the IntegrateData function to create a batch-corrected expression matrix of all cells, which allowed cells from different datasets to be integrated and analyzed together.

### Cell-type annotation and cluster marker identification

Subsequent to non-linear dimensional reduction and the projection of all cells into two-dimensional space by *t*-SNE, the cells clustered together according to common features; markers for each identified cluster were searched using the FindAllMarkers function in Seurat. The clusters were then classified and annotated based on the expression of the canonical markers of particular cell types. Clusters expressing ≥2 canonical cell-type markers were classified as doublet cells and excluded from further analysis. Pure T cells were obtained by removing cells if they met the following criteria: (1) CD3 expression (the mean expression of *CD3D*, *CD3E*, and *CD3G*) < 0.5; (2) Platelet marker expression (the mean expression of *PPBP*, *GP9*, and *PF4*) > 0.1; or (3) *CD4* expression >0.1 for CD8^+^ samples and CD8 expression (the mean expression of *CD8A* and *CD8B*) > 0.1 for CD4^+^ samples.

### DEG identification and functional enrichment

DEGs were tested using the FindMarkers function in Seurat with the parameter test.use = “wilcox” by default, and the false discovery rate (FDR) was estimated using the Benjamini–Hochberg procedure. DEGs were filtered using a minimum log_2_(fold change) of 0.5 and a maximum FDR value of 0.01. Enrichment analysis for the functions of DEGs was conducted using the Metascape webtool (www.metascape.org). Gene sets were derived from the Gene Ontology (GO) Biological Process Ontology (http://geneontology.org).

### Defining cell state scores

Cell scores were utilized to evaluate the degree to which individual cells expressed a certain predefined expression gene set^48–50^. The cell scores were initially based on the average expression of the genes from the predefined gene set in the respective cell. For a given cell *i* and a gene set *j* (*Gj*), the cell score SC*j* (*i*) quantifying the relative expression of *Gj* in cell *i* as the average relative expression (Er) of the genes in *Gj* compared to the average relative expression of a control gene set (*Gj*cont): SC*j* (*i*) = average (Er[*Gj*,*i*]) − average (Er[*Gj*cont,*i*]). The control gene set was randomly selected based on aggregate expression level bins, which yield a comparable distribution of expression levels and are greater than that of the considered gene set. The AddModuleScore function in Seurat was used to implement the method with default settings. We used RESPONSE TO INTERFERON ALPHA (GO:0035455), INFLAMMATORY RESPONSE (GO:0006954), APOPTOTIC SIGNALING PATHWAY (GO:0097190), T CELL ACTIVATION (GO:0042110), LEUKOCYTE MIGRATION (GO:0050900), four well-defined naive markers (*CCR7*, *TCF7*, *LEF1*, and *SELL*), 12 cytotoxicity-associated genes (*PRF1*, *IFNG*, *GNLY*, *NKG7*, *GZMB*, *GZMA*, *GZMH*, *KLRK1*, *KLRB1*, *KLRD1*, *CTSW,* and *CST7*), and seven well-defined exhaustion markers (*LAG3*, *TIGIT*, *PDCD1*, *CTLA4*, *HAVCR2*, *TOX,* and *CD244*) to define the IFN-α response, inflammatory response, apoptosis, activation, migration, naive-state, cytotoxicity and exhaustion score, respectively.

### TCR V(D)J sequencing and analysis

Full-length TCR V(D)J segments were enriched from amplified cDNA from 5′ libraries via polymerase chain reaction (PCR) amplification using a Chromium Single-Cell V(D)J Enrichment kit according to the manufacturer’s protocol (10× Genomics, Pleasanton, CA). Demultiplexing, gene quantification, and TCR clonotype assignment were performed using the Cell Ranger (v.3.0.2) V(D)J pipeline with GRCh38 as a reference. Briefly, a TCR diversity metric containing clonotype frequency and barcode information was obtained. Only cells with at least one productive TCR alpha chain (TRA) and one productive TCR beta chain (TRB) were retained for further analysis. Each unique TRA(s)–TRB(s) pair was defined as a clonotype. If one clonotype was present in at least two cells, cells harboring this clonotype were considered clonal. The number of cells with such pairs indicated the degree of clonality of the clonotype. T cells with prevalent TCR clonotypes were projected on *t*-SNE plots using barcode information.

### Flow cytometry

Multicolor flow cytometry was performed using the following fluorescently conjugated antibodies or reagents: anti-CD3 (OKT3), anti-CD8 (SK1), anti-TCRγδ (B1), anti-GNLY (DH2), anti-CX3CR1 (2A9-1), anti-PDCD1 (EH12.2H7), anti-T-bet (4H10), and anti-Granzyme B (QA16A02), were purchased from BioLegend (San Diego, CA), except anti-DAP12 (406288) (BD Pharmingen™, San Jose, CA) and anti-EOMES (50-4877-42) (Thermo Fisher Scientific, Waltham, MA, USA), respectively. The cells were stained with antibodies for 30 min at 4 °C for surface marker staining. For intracellular marker staining, the cells were permeabilized using a Cytofix/Cytoperm Kit (BD Biosciences), and then stained with the indicated antibodies. The cells were fixed in 0.5% formaldehyde. Data were acquired on a BD FACSCanto II flow cytometer (BD Biosciences), and further analyzed using FlowJo (Ashland, OR) Tree Star software.

### Validate the features of CD4^+^ and CD8^+^ Effector-GNLY cells

First, we gated the CD4^+^ and CD8^+^ Effector-GNLY cells through flow cytometry based on the defined markers in our scRNA-seq data (Fig. 6d). All CD4^+^ and CD8^+^ T cells (except the non-canonical T cells such as gdT and NKT cells) were used to compare with CD4^+^ and CD8^+^ Effector-GNLY cells, respectively. Then, we stained the expression levels of some cytotoxic genes (such as *GZMB* and *CX3CR1*), differentiation-associated transcription factors (*T-bet* and *EOMES*), and exhausted marker *PDCD1* and then compared the ratios of cells highly expressing these genes by flow cytometry.

### Statistics

The statistical tools, methods, and threshold for each analysis are explicitly described in the “Results” section or detailed in the figure legends or the “Materials and methods” sections.

## Supplementary information

Supplementary Information

Supplementary Table 1

Supplementary Table 2

Supplementary Table 3

Supplementary Table 4

Supplementary Table 5

## Data Availability

The data that support the findings of this study are openly available in Genome Sequence Archive of the Beijing Institute of Genomics (BIG) Data Center, Chinese Academy of Sciences at http://bigd.big.ac.cn/gsa-human under reference number HRA000190.

## Code availability

Experimental protocols and the data analysis pipeline used in our work follow the 10× Genomics and Seurat official websites. The analysis steps, functions, and parameters used are described in detail in the “Materials and methods” section. The authors declare that all relevant data and custom scripts for analyzing data are available upon reasonable request.
