# Supplementary material for: Global transcriptomic characterization of T cells in individuals with chronic HIV-1 infection
Source: Cell Discov. 2022 Mar 28;8:29. doi: 10.1038/s41421-021-00367-x (PMC8964811; doi:10.1038/s41421-021-00367-x)
Supplement: Supplementary file 1 — Supplementary Information [file 41421_2021_367_MOESM1_ESM.pdf]

Supplementary Figures and Legends

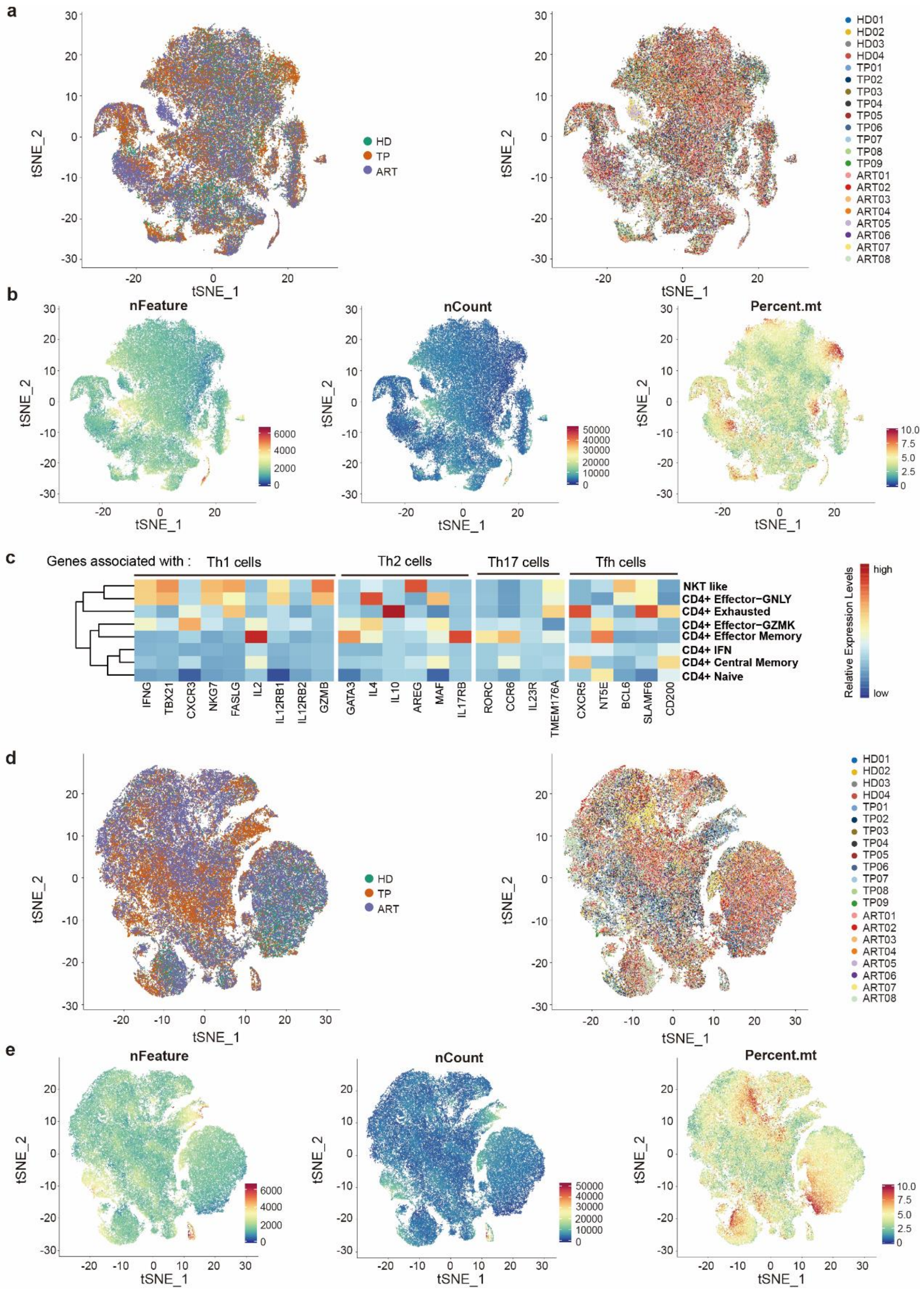

Fig. S1 Quality of clustering in CD4$^+$ and CD8$^+$ T cells for healthy donors and individuals with chronic HIV-1 infection. a t-SNE of CD4$^+$ T cells colored by condition identity (*left*) and sample identity (*right*). **b** t-SNE of gene counts (*left*), UMIs (*middle*) and percent of mitochondrial genes (*right*) in CD4$^+$ T cells. **c** Heatmap shows the expression distribution of

selected canonical T-helper markers across CD4$^+$ T subsets. The t-SNE of CD8$^+$ T cells colored by condition identity (*left*) and sample identity (*right*). **d** t-SNE of CD8+ T cells colored by condition identity (left) and sample identity (right). **e** t-SNE of gene counts (left), UMIs (middle) and percent of mitochondrial genes (right) in CD8+ T cells.

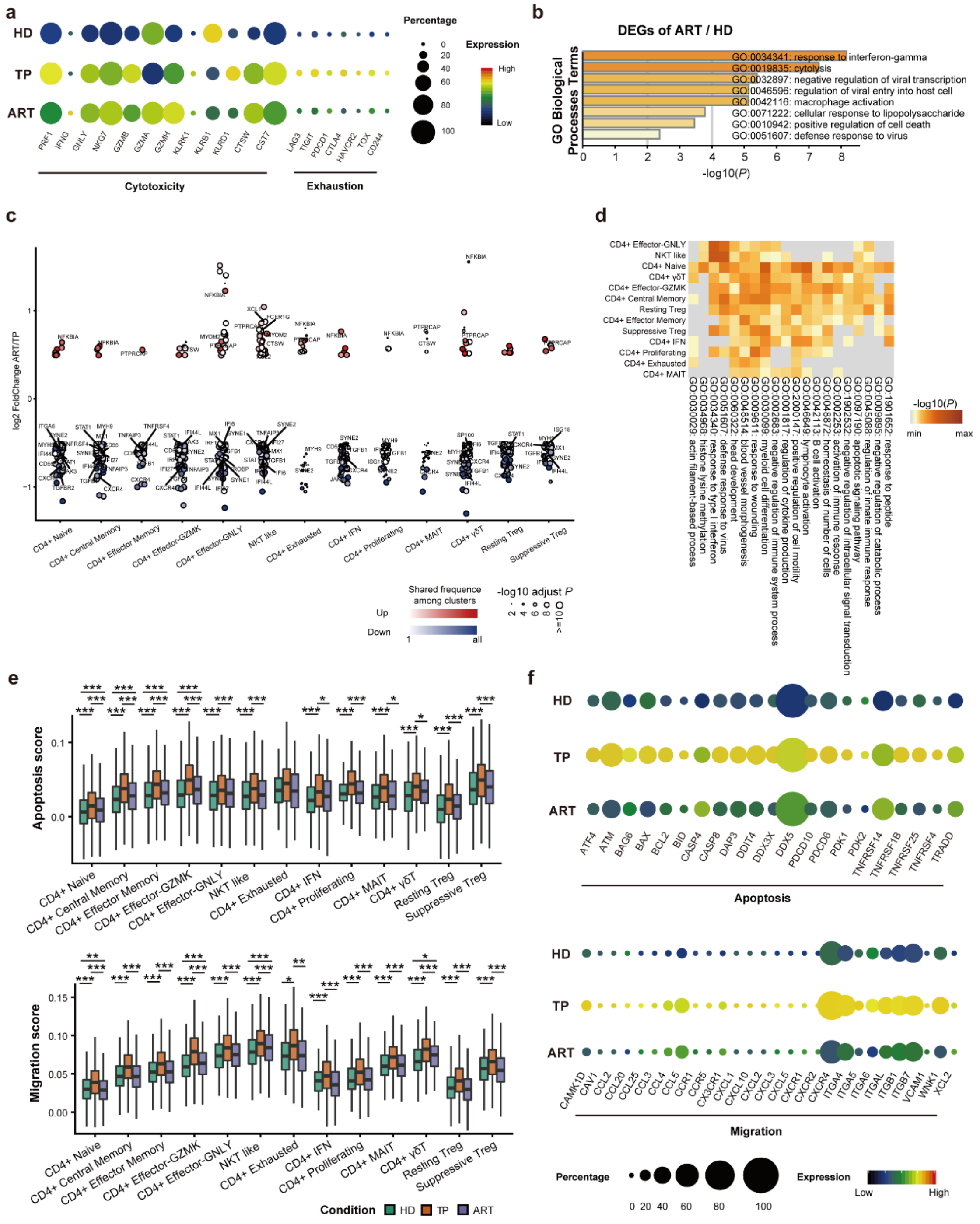

Fig. S2 Transcriptomic profiling of CD4⁺ T cells across the three conditions. a Dot plot showing expression of some well-defined cytotoxicity and exhaustion genes in CD4⁺ T cells across three conditions. The size of the circle indicates the percentage of cells expressing pathway-associated genes under each condition. The color of the circle represents the expression levels of pathway-associated genes under each condition, and color in red means a relatively high expression level and black means a relatively low expression level. **b** Gene enrichment analyses of DEGs in ARTs in comparison with healthy donors. GO terms were labeled with name and id, and sorted by -log10 (*P*) value. A darker color indicates a smaller *P*-value. **c** Log2-fold change (y axis) of DEGs between ART and TP individuals in each CD4⁺ T cell subset. Color differentiated genes with increased (red) or decreased (blue) expression in

ART condition, and point size represents statistical significance (adjusted *p* value). The transparency of the points denotes the number of comparisons in which the genes are significantly differentially expressed. **d** Heatmap shows the results of functional enrichment analyses of down-regulated DEGs from **c**. GO terms are labeled with name and ID, and the color represents −log10 (*P*) value. **e** The expression levels of two GO biological process terms across clusters derived from HD (n=4), TP (n=9) and ART (n=8) samples. Horizontal lines represent median values, with whiskers extending to the farthest data point within a maximum of 1.5 × interquartile range. $^{*}P < 0.01$; $^{**}P < 0.001$; $^{***}P < 0.0001$; two-sided unpaired Dunn's (Bonferroni) test. **f** The same to **a**, but for some genes associated with apoptosis and migration processes across the three conditions.

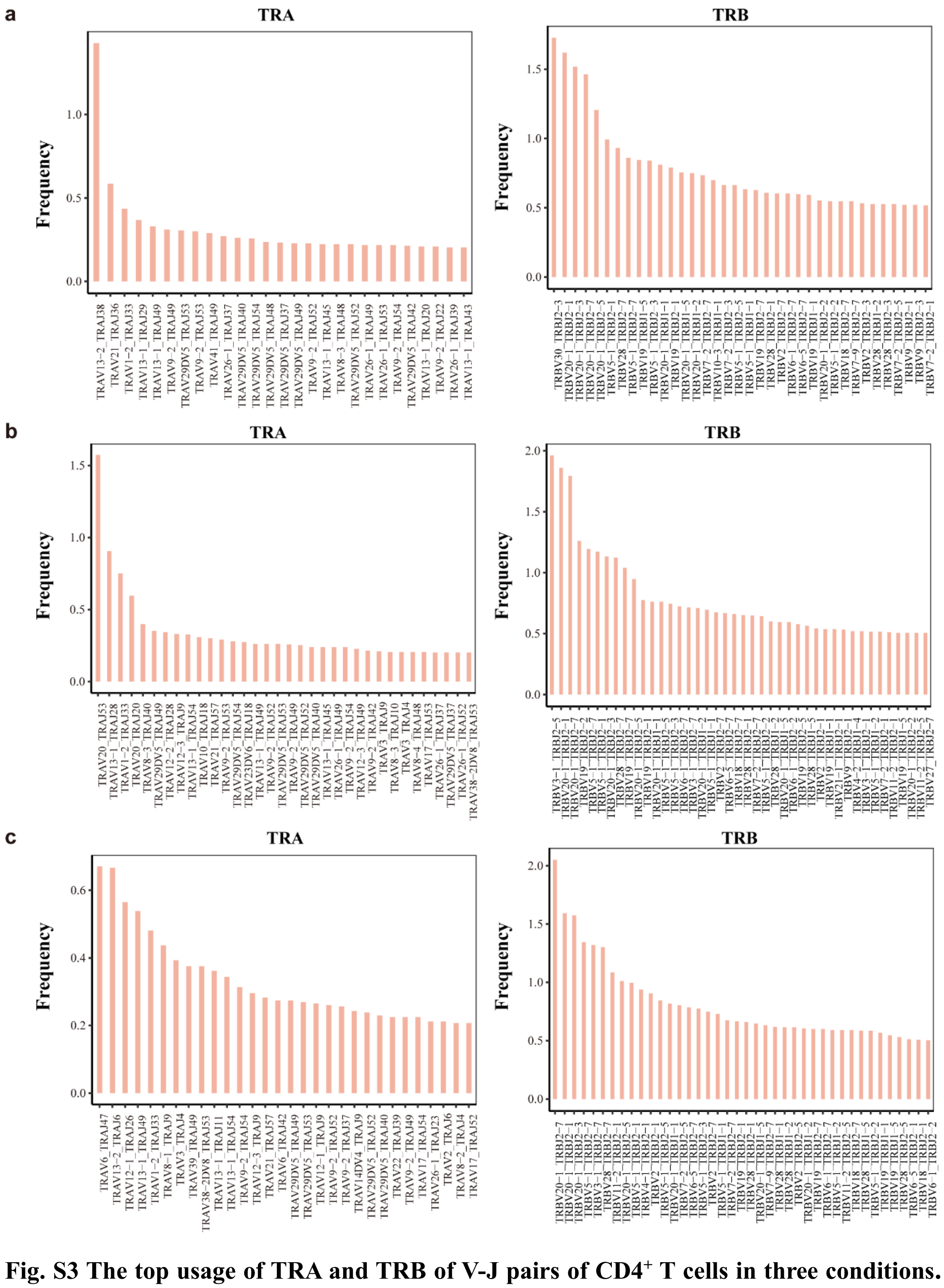

Fig. S3 The top usage of TRA and TRB of V-J pairs of CD4^{+} T cells in three conditions. For each condition, bar plots show the frequency of TRA above 0.2 and the frequency of TRB above 0.5. **a** in HD condition, **b** in TP condition and **c** in ART condition.

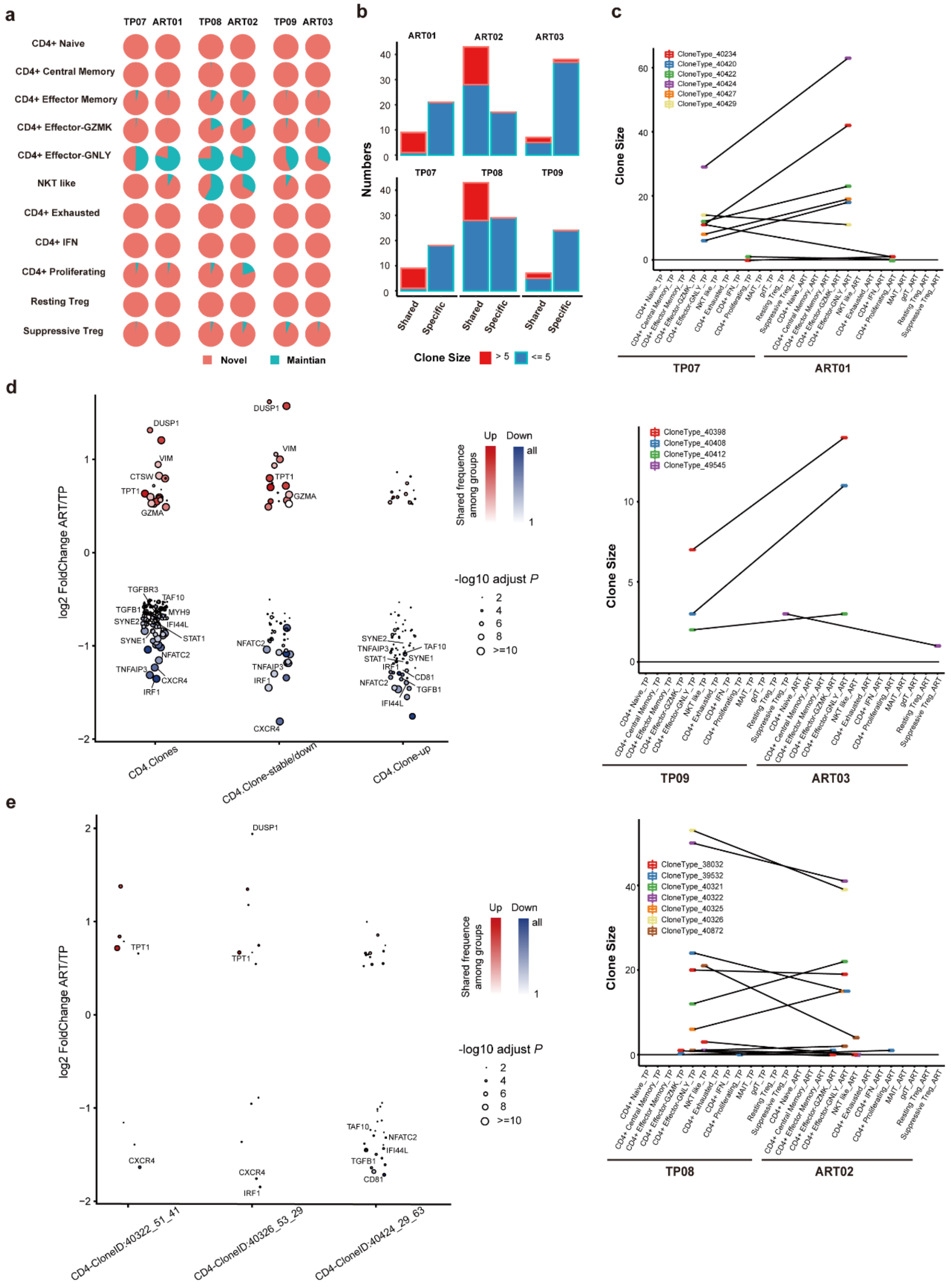

Fig. S4 Clonal maintenance of CD4^{+} Effector-GNLY cells after ART. a Pie charts showing the percentage of matched clones before and after ART among CD4^{+} T-cell subsets. **b** Distribution of match clones in big clone (clone size > 5) and small clone. **c** Changes in selected top 5 of match clones detected before and after ART. **d** DEGs of CD4^{+} clones between before and after ART. **e** DEGs of stable, contracting and expanding CD4^{+} clones between before and after ART. X-axis: clonotype id, cell numbers in TP and cell numbers after ART. Color discriminates genes with increased (red) or decreased (blue) expression in ART condition, and

point size represents statistical significance (adjusted *p* value). The transparency of the points denotes the number of comparisons in which the genes are significantly differentially expressed.

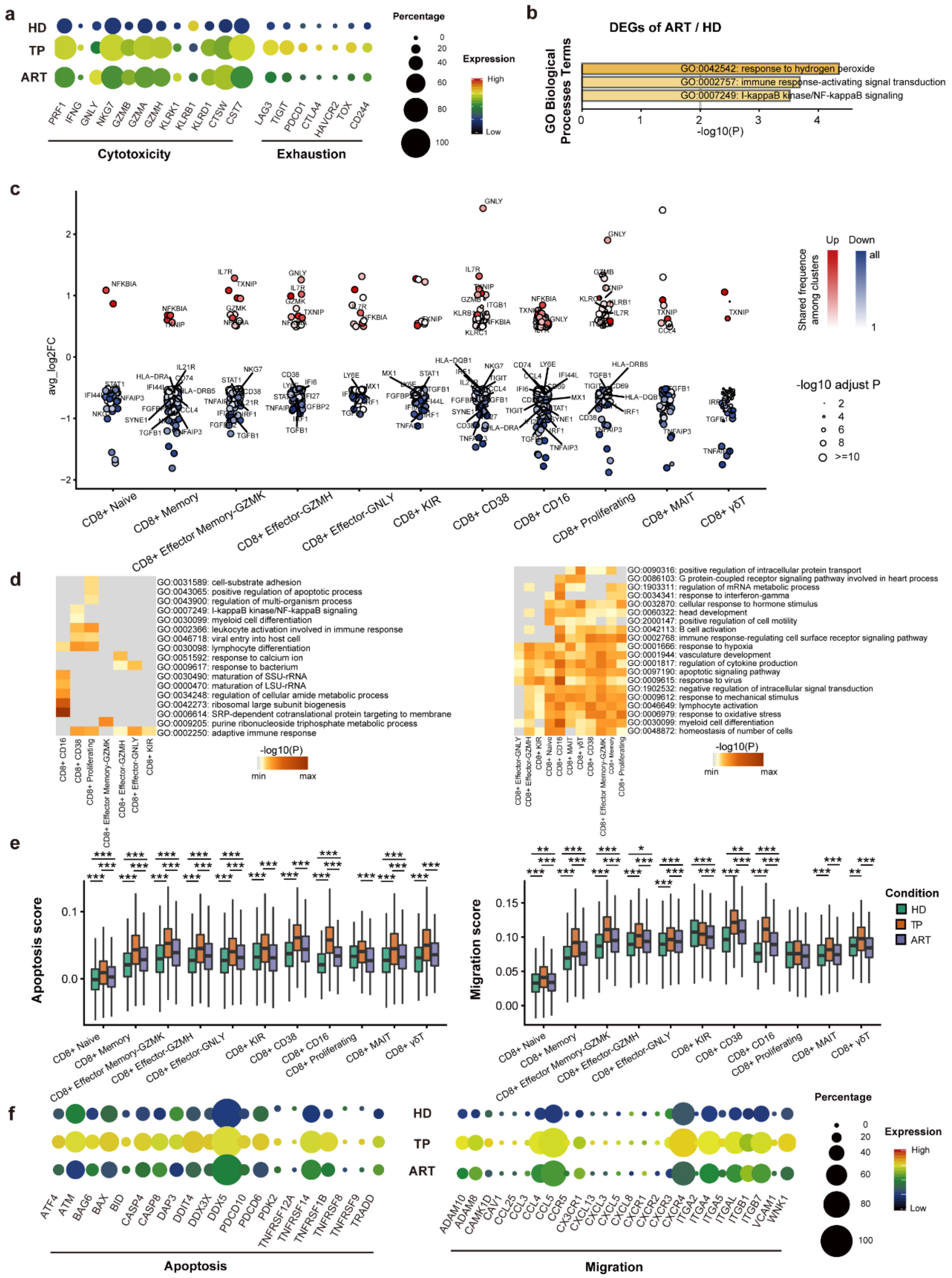

Fig. S5 Transcriptomic profiling of CD8$^+$ T cells across the three conditions. a Dot plot showing expression of some well-defined cytotoxicity and exhaustion genes in CD8$^+$ T cells across the three conditions. The size of the circle indicates the percentage of cells expressing pathway-associated genes under each condition. The color of the circle represents the expression levels of pathway-associated genes under each condition, and color in red means a relatively high expression level and black means a relatively low expression level. **b** Gene enrichment analyses of DEGs in ARTs in comparison with healthy donors. GO terms were

labeled with name and id, and sorted by -log10 (*P*) value. A darker color indicates a smaller *P*-value. **c** Log2-fold change (y axis) of DEGs between ART and TP individuals in each $CD8^+$ T cell subset. Color differentiated genes with increased (red) or decreased (blue) expression in ART condition, and point size represents statistical significance (adjusted *p* value). The transparency of the points denotes the number of comparisons in which the genes are significantly differentially expressed. **d** Heatmap shows the results of functional enrichment analyses of up-regulated DEGs (*left*) and down-regulated DEGs (*right*) from **c.** GO terms are labeled with name and ID, and the color represents −log10 (*P*) value. **e** The expression levels of two GO biological process terms across clusters derived from HD (n=4), TP (n=9) and ART (n=8) samples. Horizontal lines represent median values, with whiskers extending to the farthest data point within a maximum of 1.5 × interquartile range. *$P < 0.01$; **$P < 0.001$; ***$P < 0.0001$; two-sided unpaired Dunn's (Bonferroni) test. **f** The same to **a**, but for some genes associated with apoptosis and migration processes across the three conditions.

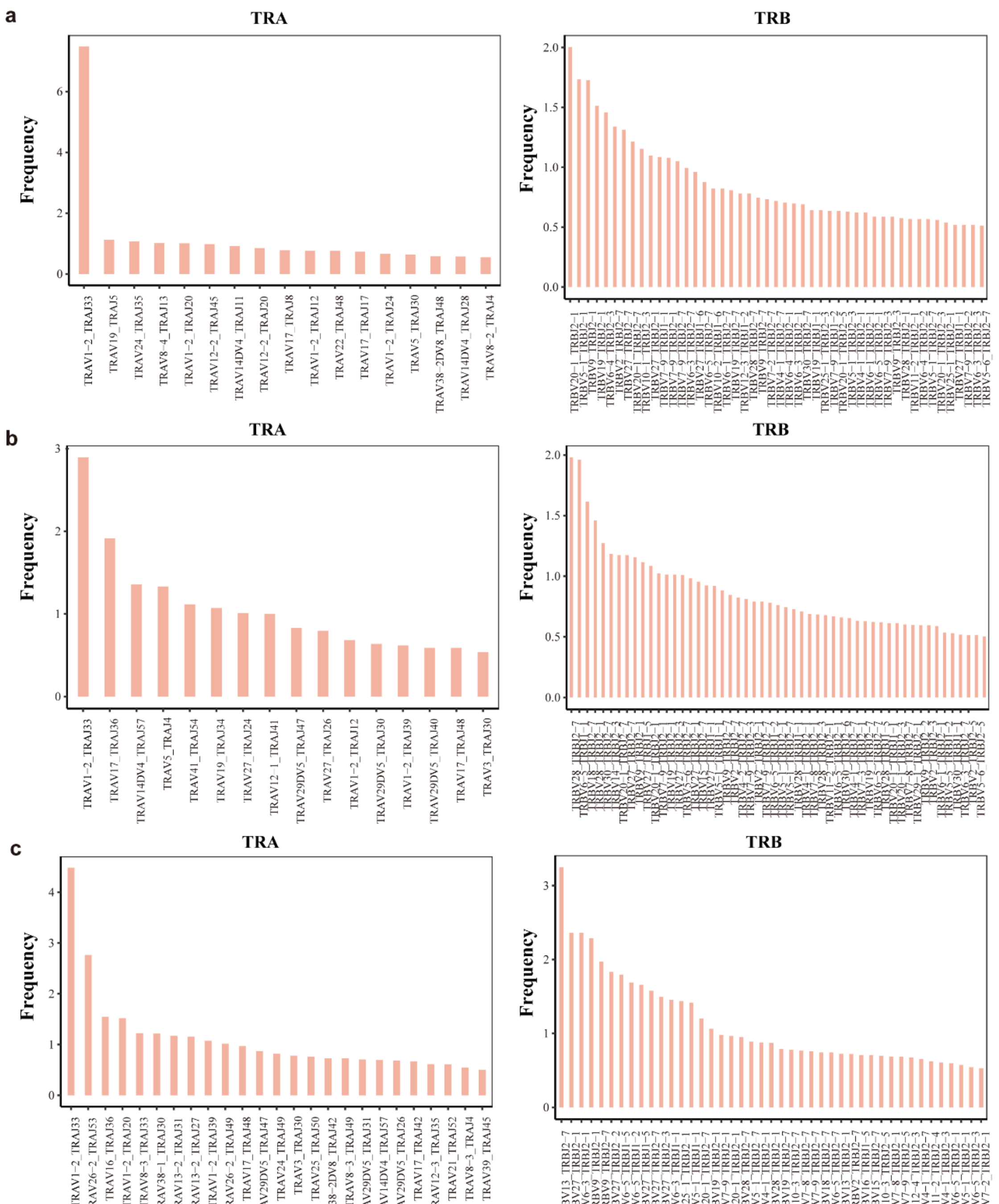

Fig. S6 The top usage of TRA and TRB of V-J pairs of CD8$^+$ T cells in three conditions. For each condition, bar plots show the frequency of TRA above 0.5 and the frequency of TRB above 0.5. **a** in HD condition, **b** in TP condition and **c** in ART condition.

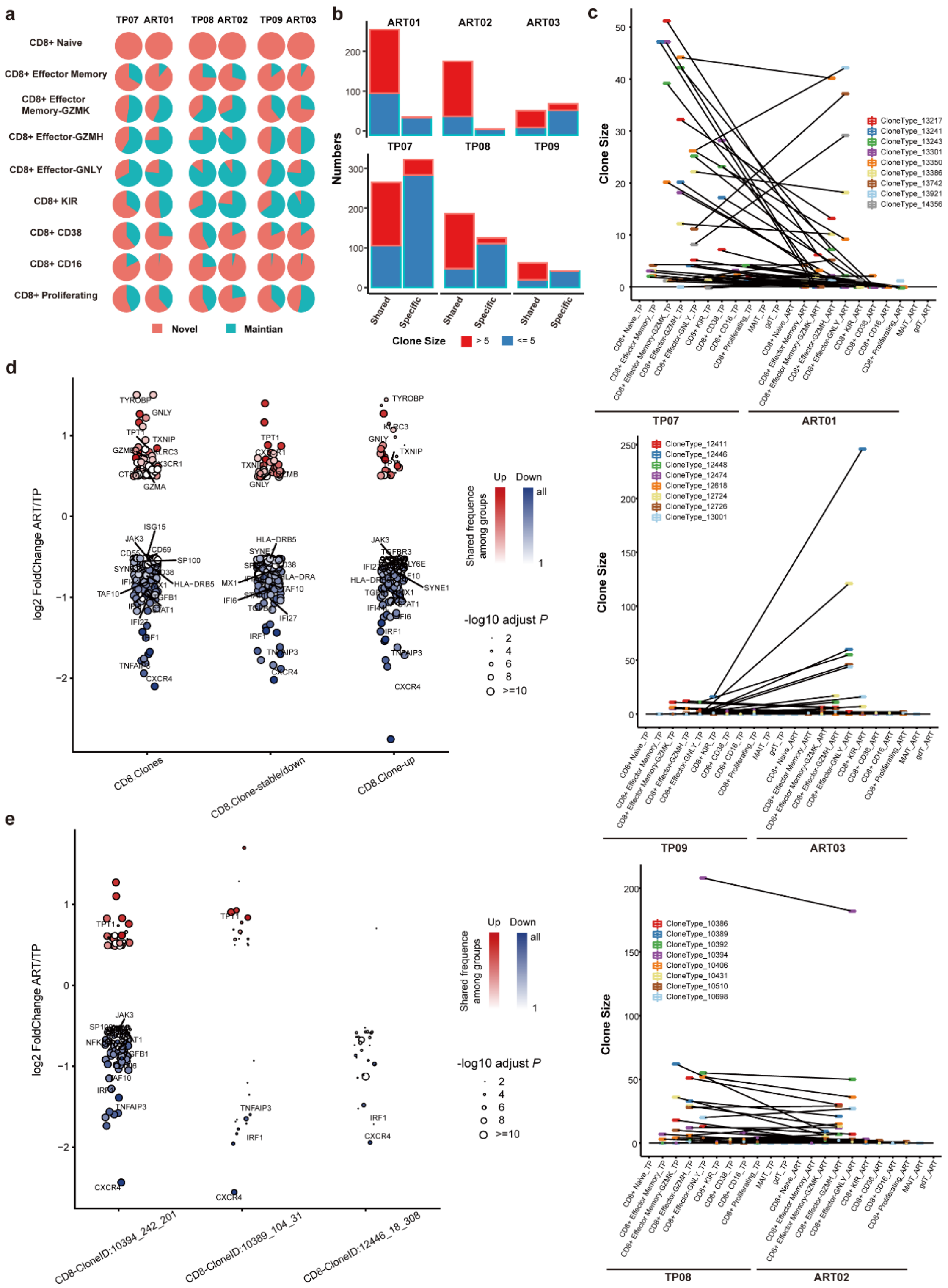

Fig. S7 Clonal maintenance of CD8⁺ Effector-GNLY cells after ART. a Pie charts showing the percentage of matched clones before and after ART among CD8⁺ T-cell subsets. **b** Distribution of match clones in big clone (clone size > 5) and small clone. **c** Changes in selected top 5 of match clones detected before and after ART. **d** DEGs of CD8⁺ clones between before and after ART. **e** DEGs of stable, contracting and expanding CD8⁺ clones between before and after ART. X-axis: clonotype id, cell numbers in TP and cell numbers after ART. Color discriminates genes with increased (red) or decreased (blue) expression in ART condition, and

point size represents statistical significance (adjusted *p* value). The transparency of the points denotes the number of comparisons in which the genes are significantly differentially expressed.

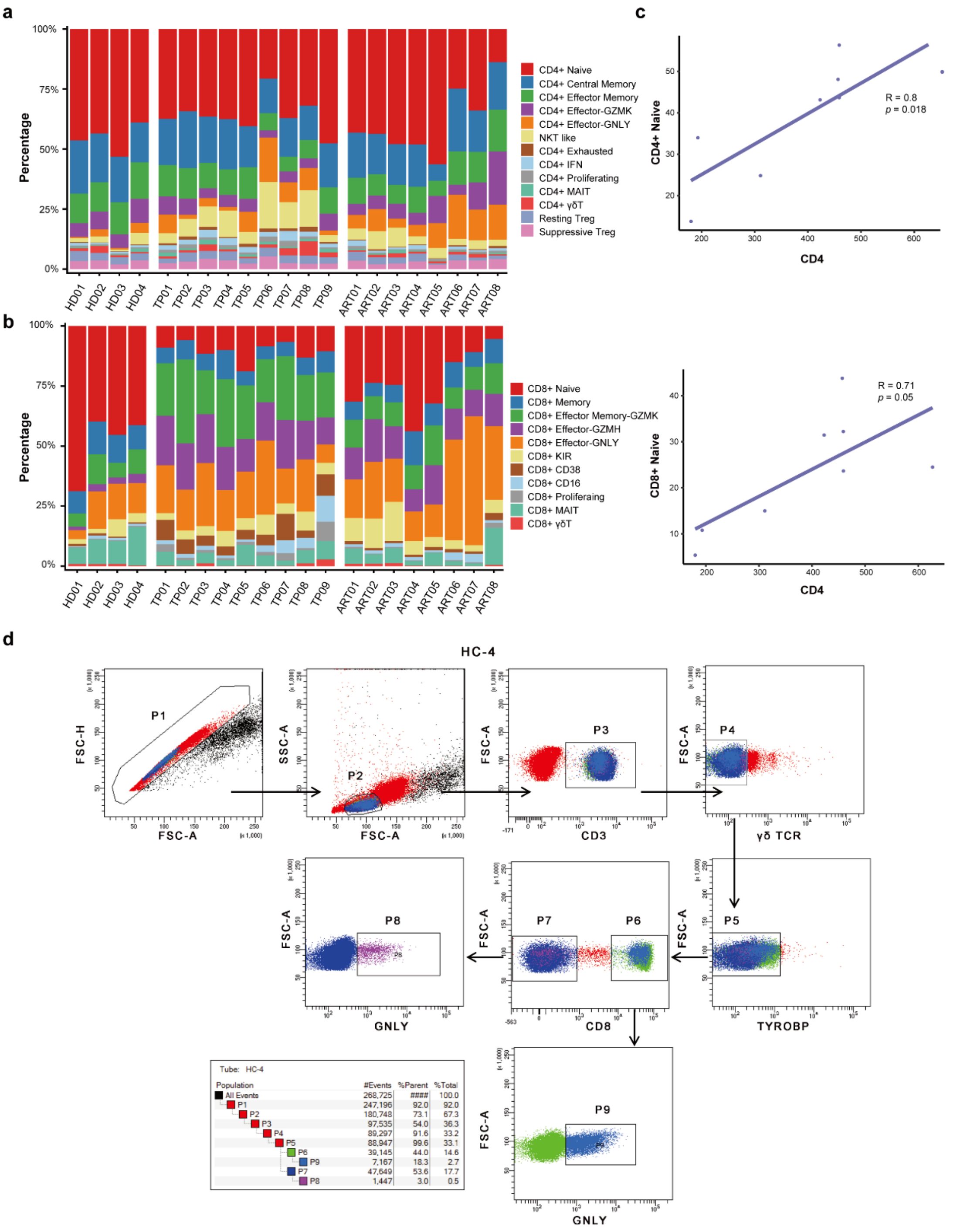

Fig. S8 Dynamic changes of CD4$^+$ and CD8$^+$ T-cell compositions. a The bar plot shows the distribution of compositions of each CD4$^+$ T-cell subset at the single sample level. **b** The bar plot shows the distribution of compositions of each CD8$^+$ T-cell subset at the single sample level. **c** Relationship between the proportions of CD4$^+$ Naïve cells (*top*) and CD8$^+$ Naïve cells (*bottom*) and the numbers of CD4$^+$ T cells in PBMCs of ARTs. **d** Gating strategy for CD4$^+$ Effector-GNLY and CD8$^+$ Effector-GNLY cells.

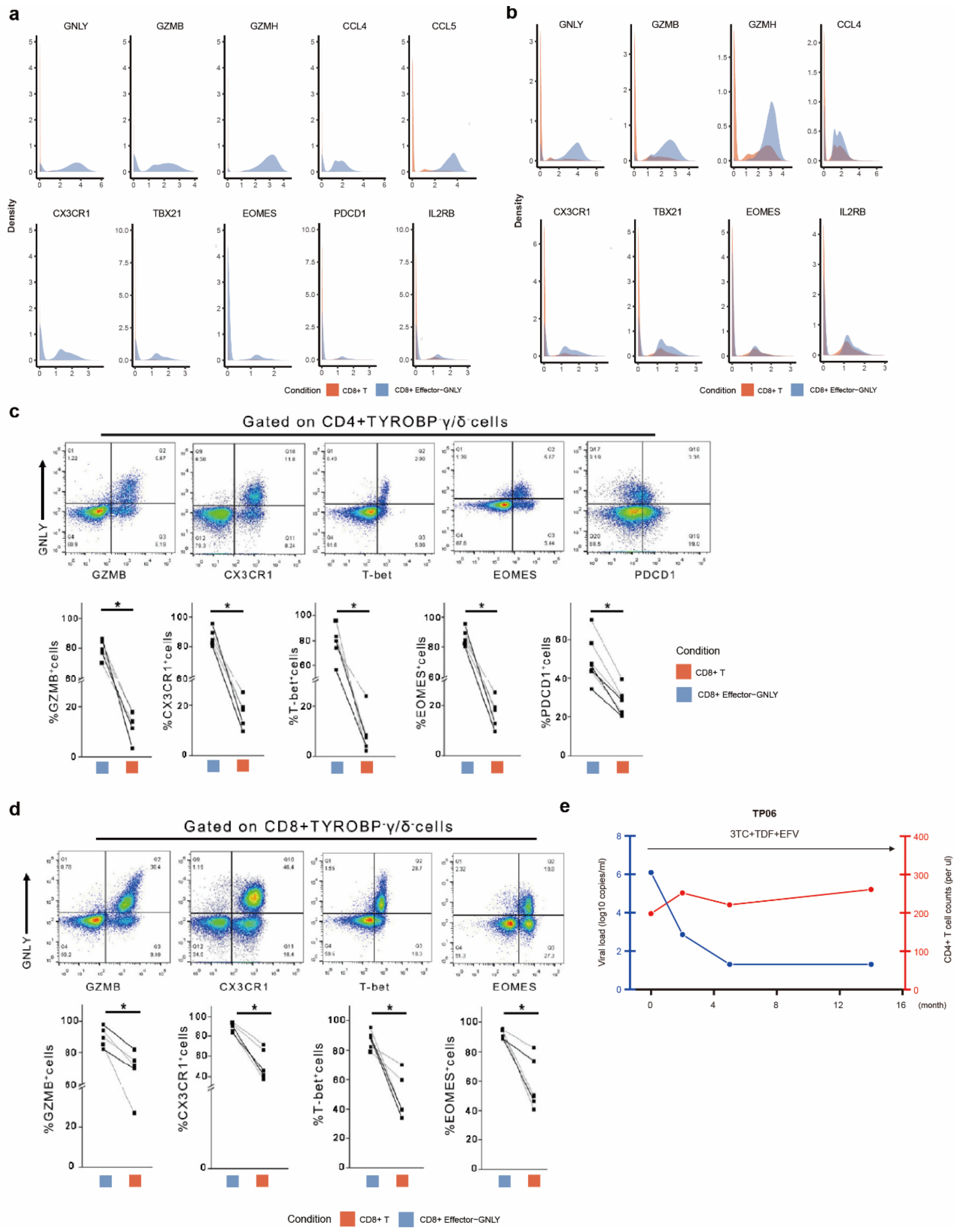

Fig. S9 Signature genes of CD4$^+$ and CD8$^+$ Effector-GNLY population. **a** Ridge plots of expression of signature genes (*GNLY*, *GZMB*, *GZMH*, *CCL4*, *CCL5*, *CX3CR1*, *TBX21*, *EOMES*, *PDCD1* and *IL2RB*) in CD4$^+$ Effector-GNLY cells. **b** Ridge plots of expression of signature genes (*GNLY*, *GZMB*, *GZMH*, *CCL4*, *CX3CR1*, *TBX21*, *EOMES* and *IL2RB*) in CD8$^+$ Effector-GNLY cells. **c** Representative FACS plots show the percentage of cells expressing *GZMB* (or *CX3CR1*, or *T-bet*, or *EOMES*, or *PDCD1*). The comparison of percentages of cells highly expressing *GZMB* (or *CX3CR1*, or *T-bet*, or *EOMES*, or *PDCD1*) between CD4$^+$ Effector-GNLY and CD4$^+$ T cells (except the non-canonical T cells such as gdT and NKT cells). For comparison, the Mann–Whitney U test was used for comparisons. *$p < 0.05$. **d** Representative

FACS plots show the percentage of cells expressing *GZMB* (or *CX3CR1*, or *T-bet*, or *EOMES*). The comparison of percentages of cells highly expressing *GZMB* (or *CX3CR1*, or *T-bet*, or *EOMES*) between $CD8^+$ Effector-GNLY and $CD8^+$ T cells (except the non-canonical T cells such as gdT and NKT cells). For comparison, the Mann–Whitney U test was used for comparisons. $*p < 0.05$. **e** Dynamic changes of HIV viral load and $CD4^+$ T cell counts in TP06 undergoing ART.
